# Managing high fiber food waste for the cultivation of black soldier fly larvae

**DOI:** 10.1038/s41538-019-0047-7

**Published:** 2019-09-02

**Authors:** Lydia Palma, Jesus Fernandez-Bayo, Deb Niemeier, Maurice Pitesky, Jean S. VanderGheynst

**Affiliations:** 1 Department of Biological and Agricultural Engineering, University of California, One Shields Avenue, Davis, CA 95616 USA; 2 Department of Civil and Environmental Engineering, University of California, One Shields Avenue, Davis, CA 95616 USA; 3 School of Veterinary Medicine, University of California, One Shields Avenue, Davis, CA 95616 USA; 4 Department of Bioengineering, University of Massachusetts, Dartmouth, MA USA

**Keywords:** Environmental biotechnology, Environmental impact

## Abstract

Increases in global human population are leading to increasing demands for food production and waste management. Insect biomass is a sustainable alternative to traditional animal feeds when insects are produced on lignocellulosic by-products. Resources high in lignocellulose have high carbon to nitrogen ratios and require nitrogen supplementation to accelerate bioconversion. Here we report on studies that examine the influence of nitrogen supplementation of almond hull-based feedstocks on black soldier fly larvae (*Hermetia illucens* L.) cultivation and composition. Decreasing carbon to nitrogen ratio from 49 to 16 increased larvae harvest dry weight, specific larvae growth, and yield by 36%, 31%, and 51%, respectively. However, the decrease in carbon to nitrogen ratio decreased larvae methionine and cysteine contents by 11% and 13%, respectively. The findings demonstrate that carbon to nitrogen ratio can be managed to enhance bioconversion of lignocellulose to larvae, but that this management approach can reduce larvae amino acid content.

## Introduction

Global human population is rapidly increasing and is expected to reach 9.8 billion by 2050.^1^ This will lead to increasing demands for food production and organic waste management. Food wastes and processing residues are one component of the organic waste stream that are currently estimated at 1.3 billion tons annually.^2^ As needs for nutritious food increase, it is critical to consider sustainable production systems that include residue reuse and valorization.

One potential use of food and agricultural wastes and processing residues is for the production of additional food resources including insects as a feed supplement.^3^ Insects are a natural source of protein, minerals, and essential amino acids and could be used to supplement poultry feed. World population increases are expected to increase demand for poultry and eggs, with demand reaching 283 million metric tons, a 50% increase, by 2050.^4^ Poultry is the most consumed protein source in the US; the price of broilers increased 17% and egg production increased 16% between 2016 and 2017.^5^ Layer hens’ diets supplemented with calcium and essential amino acids, such as methionine and cysteine, have improved laying rate and decreased feed-to-egg ratio.^6^

Black soldier fly larvae (BSFL), *Hermetia illucens* L., have the potential to play an important role in animal feed supplements and the provision of essential amino acids. BSFL cultivation systems have a relatively small ecological footprint^7^ with respect to greenhouse gases and ammonia emissions. Cultivating BSFL is less water intensive than production of several other animal feeds.^3^ In addition, the larvae are able to grow on a number of different substrates^8^ and can produce an amino acid profile comparable to soybean meal.^9^ The content of BSFL grown on vegetable waste included 7.6 g kg^−1^ methionine, 2.1 g kg^−1^ cysteine, and 28.72 g kg^−1^ calcium,^10^ while larvae fed a highly lignocellulosic substrate consisting of almond hulls included 25.3 g kg^−1^ calcium, 3.1 g kg^−1^ cysteine, and 5.2 g kg^−1^ methionine.^11^ BSFL cultivated on organic side streams also appeared to be a potential substitute for common commercially available protein sources and could replace fish meal or soybean meal without any adverse effects.^12–16^

One of the challenges of using lignocellulosic by-product streams for insect cultivation is that these feedstocks are often nitrogen poor. It is well known that nitrogen is important for the growth of microorganisms and synthesis of enzymes necessary to breakdown lignocellulose.^17^ Nitrogen supplementation of ammonium nitrate has been observed to enhance decomposition in dead vegetation containing high lignin and cellulose.^18^ In addition, treating almond hulls with urea improved digestibility and nutrient utilization for ruminant feed.^19^ Despite recent research, there remains a gap in understanding the effects nitrogen supplementation on insect cultivation. The objective of this research was to examine the impact of controlled nitrogen supplementation of high-fiber food by-products on larvae growth and composition for poultry feed. The model source of high-fiber food waste used in this study was a residue of almond production. Almond fruit consists of a kernel with an outer shell surrounded by an outer hull. California almond farming is responsible for a prodigious amount of hulls and shells; 1.93 million metric tons of hulls and 0.68 metric tons of shells in 2017 alone.^20^ The primary use of almond hulls and shells in California has historically been for livestock feed and bedding, but a recent increase in the national and global supply of livestock and dairy products resulted in reduced demand. The state lost a total of 46 dairies in 2016 and 32 dairies in 2015^21^ and with this came a reduction in demand for hulls and shells. Almond production is rising and finding alternative uses for almond by-products will be critical for sustainable almond production. The results with this model feedstock are intended to inform the engineering management of low-value, high-volume agricultural by-products for insect production.

## Results

### C/N and particle size

In general, larvae harvest dry weight and yield decreased with increasing C/N ratio (Supplementary Figs. 1 and 2). For the hull material with a maximum particle size of 6.35 mm, specific larvae growth showed a maximum at C/N of ~29 (Supplementary Fig. 3). Hull consumption increased with increasing C/N ratio (Supplementary Fig. 4) and averaged 24% of the dry weight during the 14-day experiment. Increasing C/N ratio from 16 to 45 increased hull consumption by 20% (Supplementary Fig. 4). The pH of the residual hull biomass ranged between 8.25 and 8.82 and was not significantly affected by C/N ratio (data not shown).

Stepwise regression analyses revealed that increasing the C/N ratio of hulls had a significant and negative effect on larvae harvest dry weight and larvae yield and had a significant and positive effect on hull consumption (*p* < 0.05, Table 1). Particle size had a significant and positive effect on specific larvae growth and larvae harvest dry weight. The second-order effect of C/N was significant and positive for larvae harvest dry weight.

**Table 1:** Statistical analysis of effect of C/N ratio and particle size on larvae yield, hull consumption, larvae harvest weight, and specific larvae growth

Factor	Larvae harvest dry weight (g larvae^−1^)		Specific larvae growth (g g^−1^) dry		Larvae yield (unitless)		Hull consumption (% dry basis)	
	Estimate	*p-*value	Estimate	*p-*value	Estimate	*p-*value	Estimate	*p-*value
C/N ratio	−0.00056	<0.0001^a^	−0.032	0.056	−0.0073	0.018^a^	0.014	0.013^a^
Particle size	0.00085	0.013^a^	0.090	0.013^a^			0.034	0.11
C/N ratio*C/N ratio	0.00011	0.023^a^	−0.014	0.14				

The interaction between C/N ratio and particle size was not significant for any response

^a^Indicates significant *p*-value < 0.05

Average methionine and cysteine contents of harvested larvae were 5.14 and 2.39 g kg^−1^, respectively (Supplementary Fig. 6). Increasing C/N ratio from 16 to 45 increased average methionine content by 37% and increased average cysteine content by 23%. Average calcium content of harvested larvae was 34.6 g kg^−1^ and increasing C/N ratio from 16 to 29 increased calcium by 12%, while increasing C/N ratio from 29 to 45 decreased calcium content by 11% (Supplementary Fig. 7). Larvae grown on the hull material with the larger maximum particle size had on average 4% greater calcium content, 10.5% greater methionine content and 7.4% greater cysteine content compared to larvae produced on hulls with the smaller particle size (Supplementary Figs 6 and 7).

Increasing the C/N ratio of hulls had a significant positive effect on methionine and cysteine contents of harvested larvae and increasing particle size had a significant positive effect on methionine content (*p* < 0.05, Table 2). The second-order effect of C/N was significant and negative for calcium content.

**Table 2:** Statistical analysis of effect of C/N ratio and particle size on larvae composition

Factor	Cysteine (g kg^−1^)		Methionine (g kg^−1^)		Calcium (g kg^−1^)	
	Estimate	*p-*value	Estimate	*p-*value	Estimate	*p*-value
C/N ratio	0.083	0.0002^a^	0.32	<0.0001^a^	−0.055	0.80
Particle size	0.055	0.093	0.17	0.020^a^	0.84	0.071
C/N ratio*C/N ratio	−0.016	0.094	−0.030	0.13	−0.35	0.016^a^

The interaction between C/N ratio and particle size was not significant for any response

^a^Indicates significant *p*-value < 0.05

### C/N and temperature

When larvae were cultivated at 28 °C, larvae harvest dry weight, specific growth and yield tended to decrease with increasing C/N ratio (Table 3). Increasing C/N ratio from 16 to 49 decreased average larvae harvest dry weight by 36%, average specific larvae growth by 31%, and yield by 51%. In contrast, hull consumption increased with increasing C/N ratio; as C/N ratio increased from 16 to 49, hull consumption increased by 37%.

**Table 3:** Summary of results from C/N ratio and temperature experiment

Temperature (°C)	C/N ratio	Larvae harvest dry weight (mg/larvae)	Specific larvae growth (g g^−1^) dry	Larvae yield (unitless)	Hull consumption (% dry basis)	Methionine content of larvae (g kg^−1^) dry	Cysteine content of larvae (g kg^−1^) dry
28	16	9.2 (1.1)	19.9 (2)	0.067 (0.01)	22.9 (3.6)	4.63 (0.13)	2.98 (0.06)
	32	9.7 (1.7)	22.0 (1.9)	0.057 (0.003)	29.4 (1.0)	4.91 (0.10)	3.21 (0.12)
	49	5.4 (0.5)	13.8 (0.7)	0.033 (0.003)	31.4 (2.5)	4.85 (0.22)	3.36 (0.18)
34	16	12.9 (1.7)	20.3 (2.5)	0.058 (0.005)	25.8 (1.0)	4.15 (0.12)	2.72 (0.07)
	32	10.9 (0.6)	24.2 (2.6)	0.064 (0.004)	29.1 (1.5)	4.63 (0.18)	2.90 (0.15)
	49	8.7 (0.7)	24.9 (1.6)	0.057 (0.003)	33.0 (2.7)	5.03 (0.20)	3.20 (0.10)

Standard deviations in parentheses. Three replicates for all treatments except C/N 16 at 34 °C with two replicates

In general, for a given C/N ratio, larvae harvest dry weight, specific growth, and yield were greater for larvae cultivated at 34 °C compared to 28 °C (Table 3). For larvae grown at 34 °C increasing C/N ratio from 16 to 32 increased larvae yield by 10% and increasing C/N ratio from 32 to 49 decreased larvae yield by 9%. During the 14-day experiment, hull consumption averaged 29% of the dry weight, and increasing C/N ratio from 16 to 49 increased average hull consumption by 24%.

Stepwise regression analyses revealed that increasing the C/N ratio of the initial substrate had a significant and negative effect on specific larvae growth and larvae yield and a significant and positive effect on hull consumption (*P* < 0.05, Table 4). This trend was consistent with observations from the preliminary study. Increasing temperature from 28 to 34 °C had a significant and positive effect on larvae harvest dry weight, specific larvae growth, and larvae yield. The second-order effect of C/N was significant with a negative effect for larvae harvest dry weight, specific larvae growth, and larvae yield indicating a maximum response exists for these three variables within the range of C/N ratios tested. The interaction between C/N ratio and temperature was also significant and positive for specific larvae growth and larvae yield; as C/N ratio increased the change in specific larvae growth and larvae yield was greater at 34 °C compared to 28 °C.

**Table 4:** Statistical analysis of effect of C/N ratio and temperature on larvae yield, hull consumption, larvae harvest weight, and specific larvae growth

Factor	Larvae harvest dry weight (g/larvae)		Specific larvae growth (g g^−1^) dry		Larvae yield (unitless)		Hull consumption (% dry basis)	
	Estimate	*p-*value	Estimate	*p-*value	Estimate	*p-*value	Estimate	*p-*value
C/N ratio	−0.0021	<0.0001^a^	−0.99	0.15	−0.01	<0.0001^a^	0.002	<0.0001^a^
Temperature	0.0013	0.0005^a^	2.4	0.0004^a^	0.004	0.0042^a^	0.006	0.23
C/N ratio*temperature			2.8	0.0008^a^	0.0028	<0.0001^a^		
C/N ratio*C/N ratio	−0.0014	0.033^a^	−3.6	0.0036^a^	−0.0066	0.0156^a^		

^a^Indicates significant *p*-value < 0.05

Methionine contents of harvested larvae ranged from 4.4 to 4.9 g kg^−1^ and cysteine contents ranged from 2.4 and 3.1 g kg^−1^ (Table 3). Average methionine and cysteine contents were 4% lower and 7.6% lower, respectively, for larvae grown at 34 °C compared to 28 °C. Increasing C/N ratio had a significant positive impact on methionine and cysteine content, while increasing temperature had a significant negative impact (*p* < 0.05, Table 5). The interaction between C/N and temperature was significant with a positive effect for methionine content; when larvae where cultivated at 34 °C, their methionine content increased with increasing C/N. Increasing temperature from 28 to 34 °C increased average calcium content from 21 to 25 g kg^−1^, decreased average protein content from 463 to 482 g kg^−1^, increased the average ash content from 103 to 116 g kg^−1^, and decreased average crude fat content from 71 to 40 g kg^−1^ in the harvested larvae (Supplementary Table 1).

**Table 5:** Statistical analysis of effect of C/N ratio and temperature on larvae composition

Factor	Ash (g kg^−1^)		Crude fat (g kg^−1^)		Methionine (g kg^−1^)		Cysteine (g kg^−1^)	
	Estimate	*p-*value	Estimate	*p-*value	Estimate	*p-*value	Estimate	*p*-value
C/N ratio			−1.2		0.244	0.0003^a^	0.213	<0.0001^a^
Temperature	0.70	0.007^a^	−1.59	0.007^a^	−0.091	0.0348^a^	−0.123	0.0006^a^
C/N ratio*temperature					0.168	0.0048^a^		

The second-order effect of C/N ratio was not significant for any response

^a^Indicates significant *p*-value < 0.05

## Discussion

To our knowledge, there are no published reports on the direct impact of nitrogen supplementation on the growth and composition of BSFL, particularly when grown on a high-fiber diet. Our results show that varying the C/N ratio of an almond hull-based BSFL diet had a significant impact on larvae growth, hull consumption, and amino acid composition. Larvae harvest weight and yield increased with decreasing C/N ratio. This is consistent with prior research. Upon estimating the C/N ratio of feedstocks from other studies, we can compare the mean C/N ratios of kitchen waste, banana peels, brewer’s waste, fecal sludge, and rice straw, which are predicted to be 23.7, 23.7, 16.8, 18.2, and 65.2, respectively (Table 6). Larvae grown on rice straw had prepupal weights that were 84%, 71%, 79%, and 77% lower than larvae grown on kitchen waste, banana peels, brewer’s waste, and fecal sludge, respectively.^22,23^ While C/N ratios lower than 16 could be tested, accessibility of nitrogen sources would need to be considered since nitrogen waste streams have other potential uses. Future work is needed to determine the ideal levels and sources of nitrogen for sustainable larvae production.

**Table 6:** Approximate C/N ratios of organic waste used to cultivate BSF larvae

Organic BSF feedstock	C/N ratio of feedstock	BSF prepupal harvest weight (g) dry	Sources for C/N	Source for prepupal weight
Fecal sludge	7.1–29.3	0.07	^1, 2^	^3^
Brewers spent grain	12.1–21.5	0.078	^4, 5^	^3^
Banana peels	18.4–29.0	0.055	^6, 7^	^3^
Kitchen waste	16.5–30.8	0.101	^8, 9^	^3^
Rice straw	58.7–71.7	0.016	^10, 11^	^12^
Almond hulls	72		^13^	
Amended almond hulls	29	0.0023–0.025		^13^

It is common for organisms under environmental stresses to reduce their growth rate and yield. Particularly, Carlson et al. ^24^ observed a growth rate–yield trade off in marine bacteria caused by environmental “hostility” or stressors, consisting of a combination of lack of resources, overabundance of other substances, and physical or chemical stresses.^24^ A negative relationship between bacterial growth and environmental hostility existed; as environmental hostility increased, maintenance energy required for cells increased, therefore bacterial growth decreased. The study reported that low bacterial growth was a result of insufficient supply of energy and nutrients, suggesting competition. These prior studies have been aimed at microorganisms only, yet we found similar observations in our study with respect to larvae growth and hull consumption. In all cases when larvae growth increased, hull consumption decreased. The reverse was also true; when hull consumption increased, larvae growth decreased. We speculate that this inter-relationship could be an indication of competition for resources between larvae and microorganisms or enhanced synergy between the larvae and their associated microbiota. For example, Gold et al. ^25^ reported that microbes in the larval gut and excretions can hydrolyze fibers, making the nutrients available for larval development.^25^ Another study isolated *Bacillus subtilis* from the gut of BSFL and observed that BSFL growth on chicken manure was improved for substrates that were treated with the bacterium.^26^ Monitoring the independent contributions of larvae and microorganisms on substrate decomposition would be necessary to confirm synergistic and competitive relationships on decomposition and larvae growth.

Our results for temperature effects on average larvae harvest dry weight were also consistent with prior research; we found that average dry weight was 25% greater for larvae reared on almond hulls at 34 °C compared to 28 °C. Harden and Tomberlin (2016) observed that BSF larvae reared on a pork diet at 32.2 °C had final larvae weight ~27% greater than larvae reared at 27.6 °C.^27^ The results indicate larvae production rate on almond hulls could be enhanced by production at elevated temperatures, however, work is needed to determine larvae tolerance to temperatures >34 °C.

A reduction in particle size beyond 6.35 mm did not improve larvae production or hull consumption. While a reduction in particle size could improve access to hull nutrients due to an increase in surface area for mass transfer, access would improve for both larvae and microorganisms. It is possible that any benefit provided to the larvae for nutrient access was countered by an increase in microbial growth and competition. It is also possible that the decrease in particle size reduced oxygen transport. Palma et al. ^11^ found that decreasing aeration rate from 0.36 to 0.08 mL min^−1^ g dry weight ^−1^ decreased the growth rate of larvae and consumption of hulls. In composting systems, a reduction in porosity due to factors including increasing water content and decreasing particle size has been shown to decrease biological activity and decomposition rate.^28,29^ In the current study, hulls with the larger particle size may have had higher porosity and for this reason achieved greater oxygen transport compared to hulls with the smaller particle size. These results indicate there is no benefit on larvae growth of milling the hulls to a particle size of <6.35 mm.

Our experiments revealed a significant impact of C/N ratio on larvae amino acid content. This is consistent with literature as several studies have found that the composition of BSFL varies when grown on different substrates.^10,30^ Spranghers et al. ^10^ observed methionine and cystine contents varied by 23% and 19%, respectively, when cultivated on four different substrates of chicken feed, digestate, vegetable waste, and restaurant waste.^10^ Liland et al. (2017) reported BSFL amino acid content including methionine was significantly impacted and varied by 31%, with the inclusion of varying amounts of brown algae in a processed wheat diet.^30^ Lalander et al. ^31^ also found black soldier larvae methionine content was significantly impacted by substrate, varying by 34% when cultivated on 11 different substrates and was highest for larvae fed poultry manure.^31^ Another factor that may have impacted larvae composition is differences in life cycle phases at the time of harvest for different treatments and for each experiment. Spranghers et al. ^10^ reported that larvae development time was the least when cultivated on chicken feed, taking only 12 days to achieve development, compared to 18 days when reared on restaurant waste.^10^ Lalander et al. ^31^ also reported larval development time was significantly impacted by substrate, taking 12 days to reach first prepupae for abattoir waste and 39 days for digested sludge.^31^. Another study that cultivated BSFL on chicken feed observed that the methionine content of larvae increased by 16% from 9-day to 12-day old larvae, with no change in methionine content from 6-day to 7-day larvae.^32^ Similar trends were observed for protein, ash, calcium, and crude fat content where some of the components were only affected during certain life cycle phase changes (Table 7). In the present study, all larvae were harvested at 14 days. It is possible that C/N ratio affected life cycle development of the larvae and thereby impacted larvae composition. The findings demonstrate that the production environment, particularly C/N ratio, can be managed to enhance bioconversion of lignocellulosic food waste streams, such as almond hulls, to larvae, but that this management approach can potentially reduce amino acid content in larvae.

**Table 7:** Comparison of larvae for C/N ratio and temperature study

Feedstock	Temp (°C)	C/N ratio	Life cycle phase	Protein (g kg^−1^)	Ash (g kg^−1^)	Calcium (g kg^−1^)	Crude fat (g kg^−1^)	Reference
Almond hulls	28	16–49	E-p	451.5–512.0	100.7–105.0	20.1–22.2	50.9–89.2	Present study
Almond hulls	32	16–49	E-p	455.7–474.8	111.7–119.6	22.3–27.7	27.9–51.2	Present study
Chicken feed			E-pp	400.5–403.5	85.9–90.2	27.6–30.4	277.5–282.5	^32^
Chicken feed			L-pp	401.9–406.1	95.4–96.6		239.2–244.8	^32^
Chicken feed			E-p	460.8–463.2	94.5–97.5		80.8–83.2	^32^
Chicken feed			L-p	435.9–440.1	98.8–105.2	28.2–31.8	71.7–72.3	^32^

E-pp: early prepupa (>14 days), L-pp: late prepupa, E-p: early pupa, L-p: late pupa

## Methods

### Acquisition and processing of almond hull feedstock

A pollinator variety of almond hulls and shells (hereafter referred to as hulls) was obtained from a processor in Chico, CA in June 2017 and used as larvae feedstock. Prior to acquisition, the material had been stored outdoors under a roof for ~6 months. The as-received moisture content of the material was 18% (dry basis). The composition of the hulls was determined in a prior study and reported to have a C/N ratio of 72.33 (6 g kg^−1^ total nitrogen, 434 g kg^−1^ total carbon), 0.48 g kg^−1^ (dry weight basis) methionine, 0.72 g kg^−1^ cysteine, 2.6 g kg^−1^ calcium, 143 g kg^−1^ total sugar, 560 g kg^−1^ total starch, 253 g kg^−1^ acid detergent fiber, 370 g kg^−1^ neutral detergent fiber, and 86 g kg^−1^ acid detergent lignin.^11^

The hulls were ground using a hammer mill with a 6.35 mm screen and then stored in airtight plastic bags. To achieve a smaller particle size distribution, a portion of hulls was ground using a handheld grinder to pass through a 4 mm screen. The particle size distribution of both materials was determined using a sieve shaker (AS 200 Control Sieve Shaker, Retsch Inc., Newtown, PA) by passing material through a series of six sieves with screen sizes of 0.063, 0.125, 0.25, 0.50, 1.0, and 2.0 mm. The captured sample weight on each sieve was divided by the total sample weight to get a fraction of the particles. All fractions were analyzed for moisture content gravimetrically by drying samples at 101–105 °C in a convection oven for 24 h. Two replicates of the same test were applied to both materials and the arithmetic means were used to calculate the fraction of the total solids shown in Table 8.

**Table 8:** Particle size distribution

Material	Fraction	Total solids (g g^−1^)
Almond hulls ground using hammer mill	2–6.35 mm	0.27
	1–2 mm	0.36
	0.50–1 mm	0.12
	0.25–0.50 mm	0.06
	0.125–0.25 mm	0.03
	0.063–0.125 mm	0.02
	<0.063 mm	0.00
Almond hulls ground using hammer mill followed by handheld grinder	2–4 mm	0.06
	1–2 mm	0.42
	0.50–1 mm	0.21
	0.25–0.50 mm	0.11
	0.125–0.25 mm	0.08
	0.063–0.125 mm	0.05
	<0.063 mm	0.00

### BSF larvae rearing

Larvae rearing methods are described in detail in Palma et al. ^11^ using eggs purchased from Symton Black Soldier Fly (College Station, TX, USA). Larvae were reared on chicken feed (Purina Premium Poultry Feed Layena Crumbles, Purina Animal Nutrition LLC, Shoreview, MN) at a moisture content of 500 g kg^−1^ wet basis and incubated at 28 °C. Prior research indicated larvae acclimation to almond hulls was unnecessary.^11^ For the preliminary experiment 10-day-old larvae were used and for the second experiment 5-day-old larvae were used. Prior to inoculation onto hulls, larvae were separated from feed and weighed. To have consistent larvae size in the experiments, larvae were manually separated for the preliminary experiment due to their larger size. For the second experiment, larvae were separated from feed using 1 and 2-mm sieves. Before inoculation of the bioreactors, samples of larvae were collected to assess moisture content measurement and average dry weight per larvae. Average initial weights of larvae and treatment inoculation densities are listed in Table 9.

**Table 9:** Parameters for experiments

Variables	Preliminary study	C/N ratio and temperature
Carbon to nitrogen ratio	16, 21, 25, 29, 33, 39, 45	16, 32, 49
Moisture content (g kg^−1^) wet basis	620	685
Inoculation density (g dry weight larvae)(kg dry weight hulls)^−1^	8.9	2.5
Average larvae weight in inoculum (mg dry weight) (larvae)^−1^	26	1.04
Aeration rate (mL min^−1^ g dry weight^−1^)	0.26	0.19
Incubation temperature (°C)	28	28, 34
Largest particle size (mm)	4, 6.35	6.35

### Feedstock preparation and incubations

Feedstock was amended with distilled water and nitrogen prior to incubation.^11^ Urea was used as a model nitrogen source to achieve target C/N ratios in the hull mixtures. Urea was used in these studies rather than an organic waste source of nitrogen because urea could be obtained consistently and distributed uniformly throughout the hulls. From each mix, three random samples were collected to measure pH and moisture content prior to larvae inoculation. Table 9 lists the C/N ratios, moisture content, aeration rate, inoculation density of larvae and average initial larvae size for each experiment.

A preliminary study was done to explore the impacts of C/N ratio and particle size of hulls on the following response variables: larvae growth, composition, yield and hull consumption. Seven different C/N ratios of 16, 21, 25, 29, 33, 39, and 45 were tested. Each C/N ratio tested included one bioreactor incubated with larvae and hulls at two maximum particles sizes of 4 and 6.35 mm. One bioreactor was prepared for each of the C/N ratio treatments at both particle sizes. This study was used to inform the range of C/N ratios for a second experiment that included C/N and temperature as variables.

The second experiment was designed as a 3 × 2 factorial experiment testing three levels of C/N ratio (16, 32, and 49) and two levels of temperature (28 and 34 °C). The responses included larvae growth, composition and yield, and hull consumption. The bioreactors used in the preliminary experiment were modified to increase the volume to ~1500 mL for the second experiment. Three replicate bioreactors were prepared for each treatment except for C/N of 16 at 34 °C, which only had two bioreactors due to a faulty airflow meter which restricted airflow. Larvae were cultivated in bioreactors as described in detail elsewhere.^11^ The cultivation studies ran for 14 days.

### Larvae harvest and analysis

At the end of the preliminary experiment larvae were harvested and prepared for analysis using methods described elsewhere.^11^ For the first experiment, larvae were manually separated from feedstock, counted, rinsed with distilled water and allowed to dry for 2 h in a beaker with paper towels. Larvae were stored at −20 °C and then freeze dried (Labconco 4.5 Liter Freeze Dry System, Marshall Scientific, Hampton, NH) for 3–5 days. To prepare freeze-dried larvae for analysis, they were frozen in liquid nitrogen and homogenized with an oscillating ball mill (MM400, Retsch Inc., Newtown, PA, USA). For the second experiment contents of reactors were frozen at −20 °C. Larvae were separated and counted at a later date where total larvae weight and total larvae recovered per reactor were recorded. Larvae moisture content was measured for each treatment gravimetrically as previously described. Separated larvae were stored at −20 °C and homogenized with an oscillating ball mill (MM400, Retsch Inc., Newtown, PA, USA). The homogenized larvae were freeze dried (VirTis 50-SRC-5, SP Scientific, Warminster, PA, USA) for 4 days. The calcium, dry matter, methionine, and cysteine contents were measured using methods described elsewhere.^11^ The crude fat, total crude protein, total glucose, total non-structural carbohydrates (TNC), and ash were measured at the UC Davis Analytical Laboratories (Davis, CA). Crude fat was measured using AOAC 2003.05 through a Randall modification of the standard Soxhlet extraction method.^33^ Total crude protein was measured using AOAC 990.03 and calculated from a protein factor of the nitrogen content, which was 6.25.^34^ Total glucose and TNC were measured using enzymatic hydrolysis, where the TNC is the sum of the total glucose, free fructose, and free sucrose.^35^ The ash content was measured using AOAC 942.05 through the gravimetric loss by heating the samples to 550 °C for at least 3 h.^36^ Average larvae weight at harvest was calculated by dividing the total dry weight of larvae by the number of larvae harvested. Specific larvae weight was calculated by dividing the change in larvae dry weight by the initial larvae dry weight.

### Spent hull analysis

At the end of the experiments samples of spent hulls were analyzed for moisture content and pH using methods as previously described.^11^ For both experiments moisture content and pH were analyzed at the time of larvae harvest.

### Data analysis

Larvae yield was calculated as the change in larvae dry mass per change in hull dry mass (Eq. (1)). The hull consumption was calculated as the change in hull dry mass per initial hull dry mass (Eq. (2))^11^

1 $${\mathrm{Yield}} = \frac{{{\mathrm{final}}\,{\mathrm{larvae}}\,{\mathrm{dry}}\,{\mathrm{weight}} - {\mathrm{initial}}\,{\mathrm{larvae}}\,{\mathrm{dry}}\,{\mathrm{weight}}}}{{{\mathrm{initial}}\,{\mathrm{hull}}\,{\mathrm{dry}}\,{\mathrm{weight}} - {\mathrm{final}}\,{\mathrm{hull}}\,{\mathrm{dry}}\,{\mathrm{weight}}}}$$

2 $$\% {\mathrm{Hull}}\,{\mathrm{Consumption}} = \frac{{{\mathrm{initial}}\,{\mathrm{hull}}\,{\mathrm{dry}}\,{\mathrm{weight}} - {\mathrm{final}}\,{\mathrm{hull}}\,{\mathrm{dry}}\,{\mathrm{weight}}}}{{{\mathrm{initial}}\,{\mathrm{hull}}\,{\mathrm{dry}}\,{\mathrm{weight}}}} \ast 100\%$$

Results from both experiments were analyzed by step-wise regression using JMP IN v.14.0.0 (SAS Institute, Cary, NC, USA) to determine if factors had significant effects on larvae yield, hull consumption, larvae dry weight at harvest, specific larvae growth, and final larvae composition. For the preliminary experiment factors were C/N ratio and particle size. For the second experiment factors were C/N ratio and temperature. Responses were analyzed using the following equation:

3 $$E\left\{ Y \right\} = \beta _0 + \beta _1X_1 + \beta _2X_2 + \beta _{12}X_1X_2 + \beta _{11}X_1^2$$

where *E*{*Y*} is the expected value of the response variable, *X*_1_ is the coded level (ranging from −3 to 3 or −1 to 1) for the C/N ratio, *X*_2_ is the coded level for high and low (−1, 1) for the second factor (particle size or temperature), *β*_1_ is the parameter estimate for C/N ratio, *β*_2_ is the parameter estimate for the second factor, *β*_12_ is the parameter estimate for the interaction between C/N ratio and second factor, and *β*_11_ is the parameter estimate representing second-order effects of C/N ratio.

### Statement of human and animal rights

Experiments were performed only on insect larvae.

### Reporting summary

Further information on research design is available in the Nature Research Reporting Summary linked to this article.

## Supplementary information

Supplemental Material

Reporting Summary

## Data Availability

The authors declare that all data supporting the findings of this study are available within the paper and its supplementary information files.

## Supplementary information

**Supplementary Information** accompanies the paper on the *npj Science of Food* website (10.1038/s41538-019-0047-7).
