# Supplementary material for: Managing high fiber food waste for the cultivation of black soldier fly larvae
Source: NPJ Sci Food. 2019 Sep 2;3:15. doi: 10.1038/s41538-019-0047-7 (PMC6718667; doi:10.1038/s41538-019-0047-7)
Supplement: Supplementary file 1 — Supplemental Material [file 41538_2019_47_MOESM1_ESM.pdf]

Supplemental Material

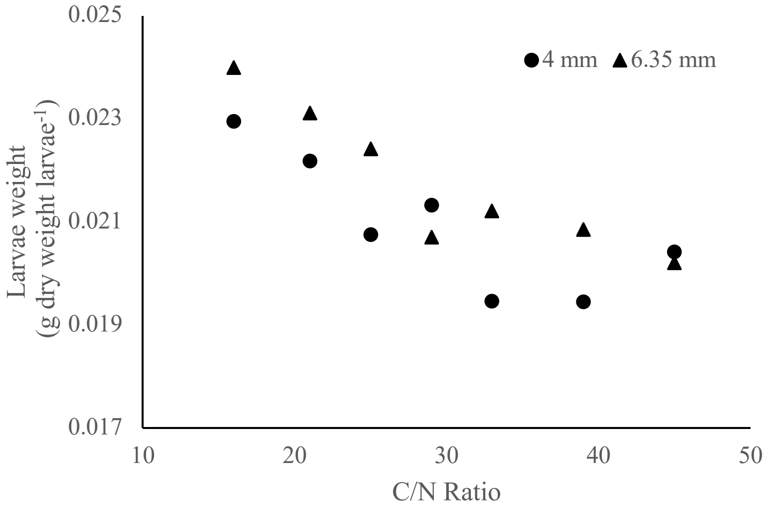

Supplementary Figure 1. Larvae harvest dry weight for growth on hulls at two different particle sizes

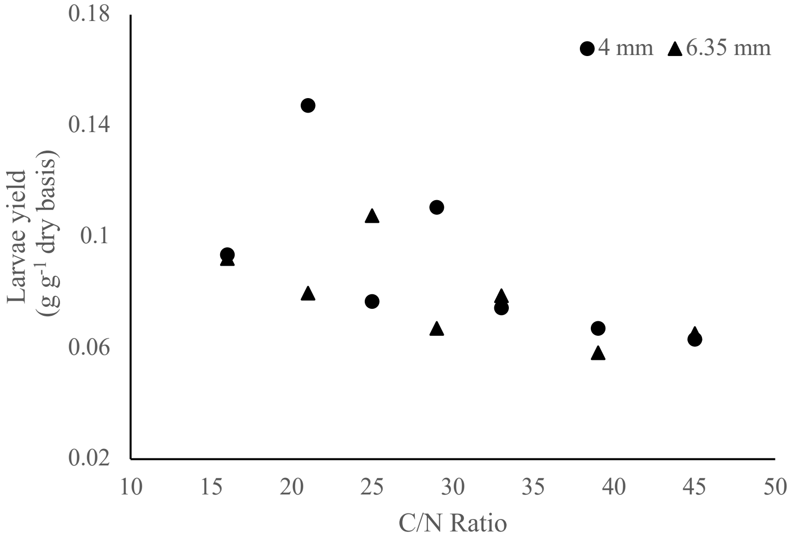

Supplementary Figure 2. Larvae yield for growth on hulls at two different particle sizes

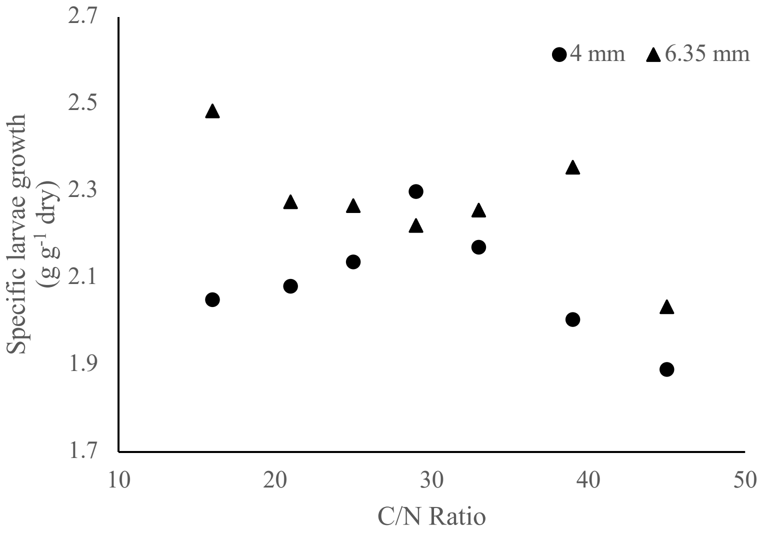

Supplementary Figure 3. Specific larvae growth for growth on hulls at two different particle sizes

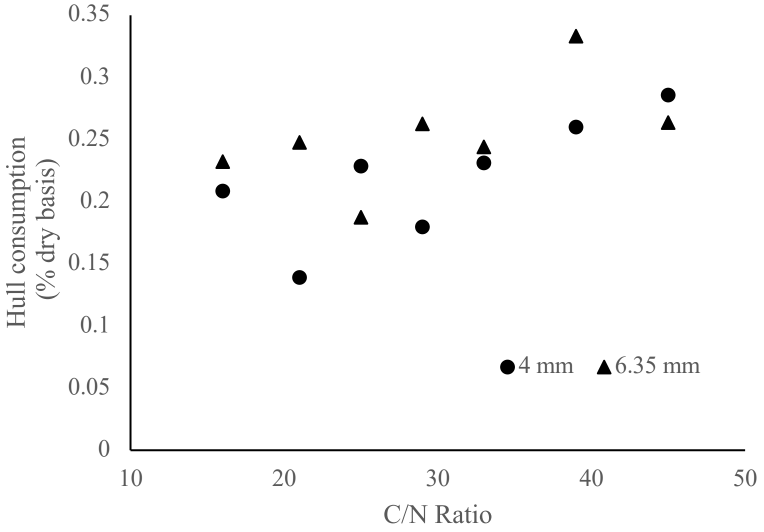

Supplementary Figure 4. Hull consumption for growth on hulls at two different particle sizes

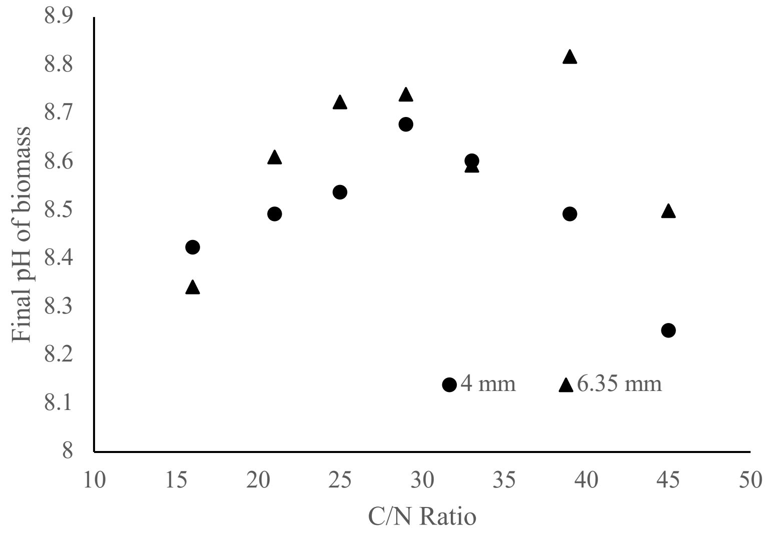

Supplementary Figure 5. Residual hull biomass pH for larvae growth on hulls at two different particle sizes

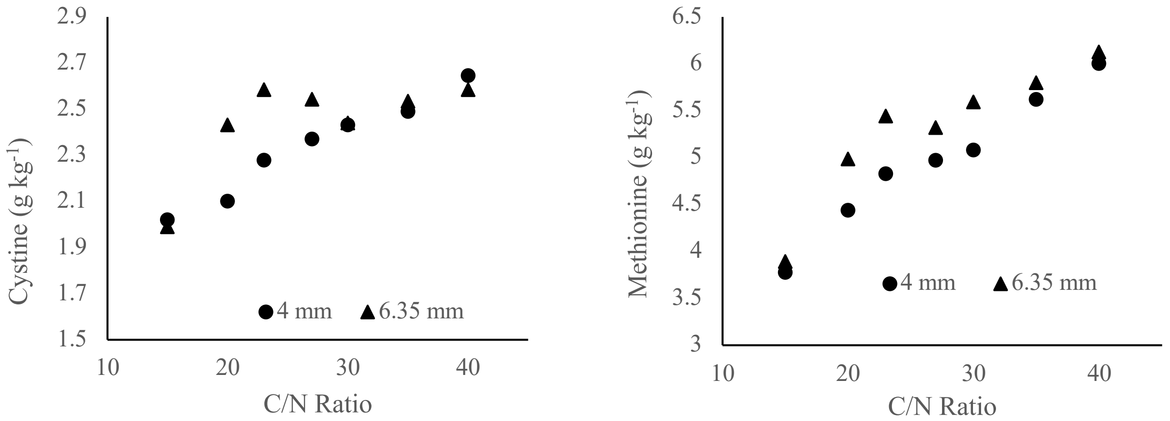

Supplementary Figure 6. Larvae amino acid composition for growth on hulls at two different particle sizes

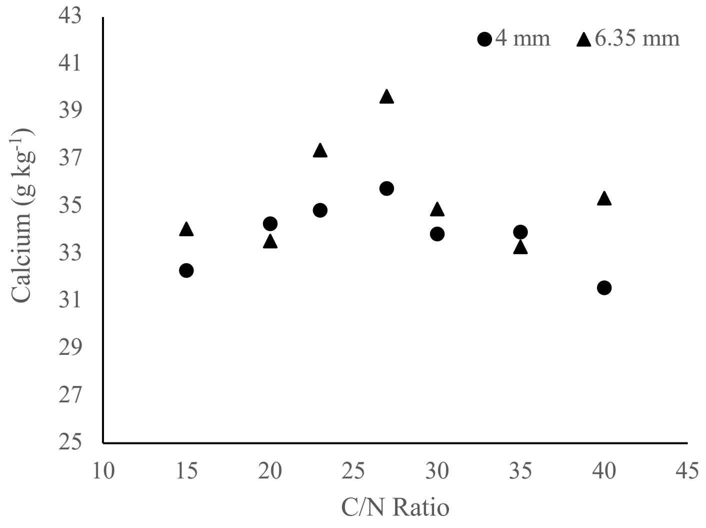

Supplementary Figure 7. Larvae calcium composition for growth on hulls at two different particle sizes

Supplementary Table 1. Larvae composition for C/N ratio and temperature experiment

Temperature (°C)	C/N ratio	Calcium ($g\ kg^{-1}$)	Protein ($g\ kg^{-1}$)	Ash ($g\ kg^{-1}$)	Crude fat ($g\ kg^{-1}$)
28	16	20.9	512.0	101.9	89.2
	32	22.2	451.5	100.7	74.0
	49	20.1	482.6	105.0	50.9
34	16	27.7	455.7	119.6	39.4
	32	22.3	474.8	111.7	51.2
	49	26.0	457.7	118.0	27.9

Three replicates combined for analysis except C/N 16 at 34°C with 2 replicates.
